# A Multi-Modal Wireless Sensor System for River Monitoring: A Case for Kikuletwa River Floods in Tanzania

**DOI:** 10.3390/s23084055

**Published:** 2023-04-17

**Authors:** Lawrence Mdegela, Yorick De Bock, Esteban Municio, Edith Luhanga, Judith Leo, Erik Mannens

**Affiliations:** 1 Department of Computer Science, University of Antwerp-imec IDLab, Sint-Pietersvliet 7, 2000 Antwerpen, Belgium; 2 The Nelson Mandela African Institution of Science and Technology, Arusha P.O. Box 447, Tanzania; 3 i2CAT Foundation, 08034 Barcelona, Spain; 4 Carnegie Mellon University Africa, Kigali P.O. Box 6150, Rwanda

**Keywords:** wireless sensors, multi-featured data, machine learning, river floods, flood detection

## Abstract

Reliable and accurate flood prediction in poorly gauged basins is challenging due to data scarcity, especially in developing countries where many rivers remain insufficiently monitored. This hinders the design and development of advanced flood prediction models and early warning systems. This paper introduces a multi-modal, sensor-based, near-real-time river monitoring system that produces a multi-feature data set for the Kikuletwa River in Northern Tanzania, an area frequently affected by floods. The system improves upon existing literature by collecting six parameters relevant to weather and river flood detection: current hour rainfall (mm), previous hour rainfall (mm/h), previous day rainfall (mm/day), river level (cm), wind speed (km/h), and wind direction. These data complement the existing local weather station functionalities and can be used for river monitoring and extreme weather prediction. Tanzanian river basins currently lack reliable mechanisms for accurately establishing river thresholds for anomaly detection, which is essential for flood prediction models. The proposed monitoring system addresses this issue by gathering information about river depth levels and weather conditions at multiple locations. This broadens the ground truth of river characteristics, ultimately improving the accuracy of flood predictions. We provide details on the monitoring system used to gather the data, as well as report on the methodology and the nature of the data. The discussion then focuses on the relevance of the data set in the context of flood prediction, the most suitable AI/ML-based forecasting approaches, and highlights potential applications beyond flood warning systems.

## 1. Introduction

**Figure 1:**
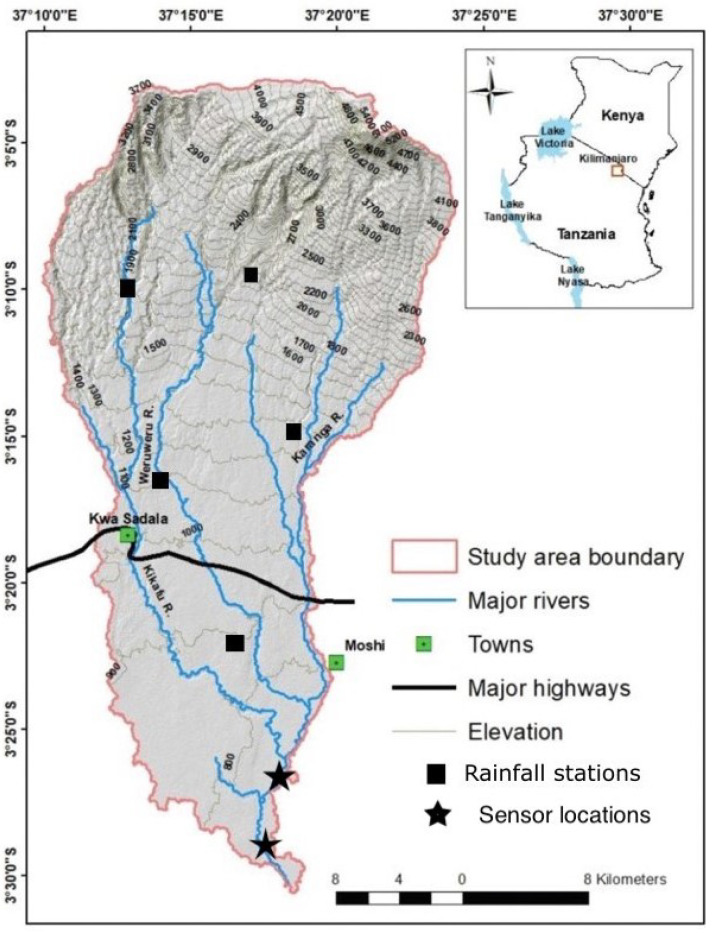
Lower Kikuletwa sub-catchment.

The lower Kikuletwa sub-catchment is an area of about 6657 km^2^ located in the north-western part of the Pangani River basin, north-east of Tanzania, south of Mount Kilimanjaro (see Figure 1). River Kikuletwa has been subject to several flood events throughout its history, the main victims of the floods being the villages along the river, especially the lower parts downstream. In April 2020, according to flood-list (https://floodlist.com/africa/tanzania-floods-arusha-kilimanjaro-april-2020) (accessed on 18 January 2023), more than 2700 households in the Moshi district were swept away by floods after a period of heavy rainfall leaving dozens homeless and destroying important infrastructures. Although natural disasters such as floods are inevitable, communities residing in flood-prone areas are often ill-equipped to mitigate their impacts. One straightforward strategy to reduce the adverse effects of floods is to improve existing flood warning mechanisms. Such measures would enable the prompt evacuation of vulnerable populations, the timely relocation of valuable assets, and ensure better preparedness to deal with the aftermath of the event. The significance of reliable and accurate flood warning mechanisms is paramount in saving lives, reducing property damage, and enhancing the overall resilience of communities facing such challenges [1]. Thus, it is crucial that appropriate steps are taken to prioritize the development and implementation of effective flood warning systems. Among the most successful technologies to identify, classify, and predict such flooding events, are Machine Learning (ML) models [2,3,4]. Given an appropriate data set (large enough, representative, multi-featured, etc.) these models can significantly outperform traditional approaches [5]. In this sense, data sets are the foundation of training, evaluating, and bench-marking machine learning models, and they play a key role in the advancement of the field [6].

**Figure 2:**
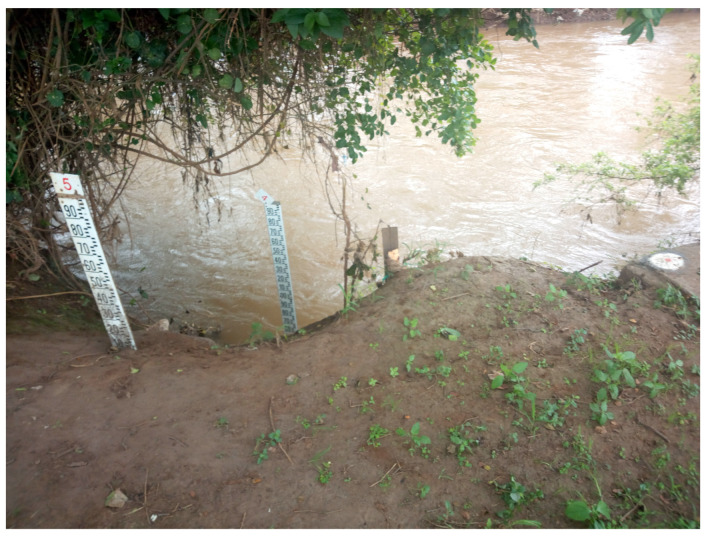
Water level staff gauges.

The scarcity of data remains one of the critical challenges in improving the accuracy of flood warning systems, especially in areas with poorly gauged basins [7]. Manual water level staff gauges, as shown in Figure 2, are currently being utilized to monitor and to record river levels in some parts of the river. However, the limitations of these manual gauges can potentially impact on the reliability and accuracy of the data collected. Addressing this issue is crucial in enhancing the effectiveness of flood warning systems and reducing the adverse impact of floods on vulnerable communities. However, despite the fact that staff gauges are cheap to implement, there are several challenges that are associated with this type of monitoring when using them for river level measurement [8]. Some of the challenges include errors due to bad angle reading, turbulence, or dirt on the scale due to debris.

One of the foremost challenges associated with monitoring rivers in flood-prone areas, such as the Kikuletwa river, is the inability to obtain high flow readings during extreme flood events. This is often due to the dangerous conditions that arise during such events, preventing human intervention for data collection until safe conditions prevail. However, the high flow readings are critical for determining the threshold values that define the levels at which flood warnings will be issued. This emphasizes the significance of acquiring accurate and reliable data during these high flow events in order to improve the efficacy of flood warning systems. Failure to obtain such data can impede the development of effective flood warning models, and compromise the ability to mitigate the impact of flood events on vulnerable communities.

In order to deal with these challenges, in this article we present a wireless sensor-based river monitoring system that enables robust, autonomous, and large-scale data gathering in a low-cost manner without the need for human intervention.

The deployment of wireless sensors in poorly gauged rivers in developing countries is a significant contribution to flood monitoring and disaster response planning. This work proposes an efficient and cost-effective approach for collecting real-time, multi-feature data on river levels, which can facilitate the establishment of effective flood warning systems and aid with disaster response efforts. Additionally, the deployment and processes involved in this study can offer insights into designing superior sensors for similar environments.

The continuous monitoring of river levels with wireless sensors enables the system to respond quickly to changes in water levels, providing timely and accurate information that can be used to issue flood warnings and make informed decisions regarding evacuation and relief efforts [9].

Moreover, the deployment of wireless sensors in poorly gauged rivers can help bridge the gap in data on flood patterns and extreme events, providing a means to model floods based on extreme levels. This approach offers an alternative to traditional methods that relies on human monitoring and data collection, which may be impractical during flood events.

The in-field deployed monitoring system proves to be successful in producing continuous data series of river water level and weather data (e.g., river depth, rainfall, wind, etc.), and is able to export the data in a standard format for storage and interpretation. Finally, we report and characterize the obtained data set and discuss its relevance for data-feeding of early warning and forecasting systems based on AI/ML models.

## 2. Background and State of the Art

The implementation of sensor-based river monitoring systems has gained prominence in recent years due to the increasing need for accurate and real-time data for effective water management and flood prediction [10]. In the last decade, reports show that there has been an increase in the frequency of flood events all over Tanzania, with almost the same impacts each year; loss of lives, infrastructures, and other societal systems (Burundi and Tanzania—Floods Leave Homes Destroyed, Hundreds Displaced. https://floodlist.com/africa/burundi-tanzania-floods-late-february-2021) (accessed on 24 January 2023), (Tanzania—Severe Flooding in Mtwara Region After Torrential Rainfall, https://floodlist.com/africa/tanzania-flood-mtwara-january-2021 (accessed on 24 January 2023), Tanzania—12 Killed in Dar Es Salaam Flash Floods. https://floodlist.com/africa/tanzania-daressalaam-floods-october-2020) (accessed on 24 January 2023). Worryingly, with the current climate change, such events are expected to be more frequent. The persistence of such events and their subsequent impacts are attributed mainly to the limited knowledge regarding their occurrence. This is partly due to data scarcity arising from poorly gauged or ungauged catchments. To effectively prepare for future hazards, it is imperative to have a better understanding of past and current events. Unfortunately, this is not the case in Tanzania as the current status quo is deficient in providing adequate data and knowledge on the subject. As such, there is a need to prioritize efforts towards addressing this knowledge gap to enhance preparedness and to mitigate the adverse effects of natural disasters such as river floods.

Kikuletwa River in Northern Tanzania is one of the many rivers that face challenges in terms of flash floods, which cause significant damage to infrastructure and agriculture, and cause loss of life. Implementing a sensor-based monitoring system is critical for mitigating the impact of floods and ensuring sustainable water management practices [11]. The traditional river monitoring systems employed, manual staff gauges, have shown little success, particularly when it is time to provide early warnings for floods. The use of sensors to automatically monitor different river characteristics is one of the promising mechanisms in overcoming this challenge. With enough and quality data, reliable and accurate early warning systems can be developed and deployed. Sensor-based monitoring systems use a network of sensors placed along the river to collect data on water levels, discharge, and rainfall, among other parameters [12]. These data are transmitted in real-time to a central server for analysis and integration with other data sources, such as satellite imagery and meteorological data [13]. The use of sensors provides a cost-effective and efficient solution compared to traditional manual monitoring methods [14]. The collected data can be used to generate early warning systems for flood events, allowing communities to prepare and evacuate in advance. In addition to providing real-time data for flood prediction and water management, sensor-based monitoring systems also offer several other benefits. For instance, they provide a continuous and comprehensive data record that can be used for scientific research and policy-making [15].

Further evidence from the literature shows that sensor-based monitoring systems are highly effective in providing real-time data for flood prediction and water management. This is the case, for example, of the water quality management project reported in [12], where different types of wireless sensor nodes were employed to obtain continuous real-time data for water quality control. Similarly, in projects such as [16], a wireless sensor network was installed in river Sitnica in Kosovo for real-time surface water quality control. More examples can be found in [10,12,17,18,19]. There are also examples of works that use such networks for early warning systems. For example, in Argentina, a sensor-based early flood detection and warning system was developed by Libelium in 2018; this was after several costly and unsuccessful efforts to solve a persistent flood problem in some villages [20]. However, their focus was mainly on flood detection, solely based on the river level thresholds. In contrast, our work generates exportable monitoring data to systematically feed AI/ML algorithms, and to aim for a more sophisticated flood warning system that includes multi-feature flood forecasting. In this sense, we would rather follow the trend that has been paved by other works such as [2,4,21,22]. Unlike most of these deployments, in a developing country such as Tanzania, there are several challenging factors such as cost (i.e., both in terms money and human resources) and available infrastructure that also need to be considered in the design. It is in this view that our work presents a low-cost, self-powered, automated monitoring system that aims to solve such challenges through a standalone system that uses low-cost devices and that removes human dependency.

This work focuses on designing and deploying a multi-model wireless sensor system for near-real-time river monitoring. The system is intended to collect a multi-feature data set that is suitable for river anomaly detection and AI/ML prediction tasks. Six parameters—current hour rainfall (mm), previous hour rainfall (mm/h), previous day rainfall (mm/day), river level (cm), wind speed (km/h), and wind direction—are aggregated, summarized, and visualized. River level data, in particular, can be utilized to accurately establish thresholds for predicting river anomalies in poorly gauged basins. The main contributions are the following:

- A multi-model implementation which supports the collection and aggregation of six weather parameters to complement existing local weather stations in building resilience to extreme weather events prediction.
- The development of a continuous data set on river characteristics which can be used in the context of improving flood resilience and water management.
- A mechanism to define river thresholds, which are a key input in anomaly detection ML models for floods.

## 3. Methodology and Data Sourcing

**Figure 3:**
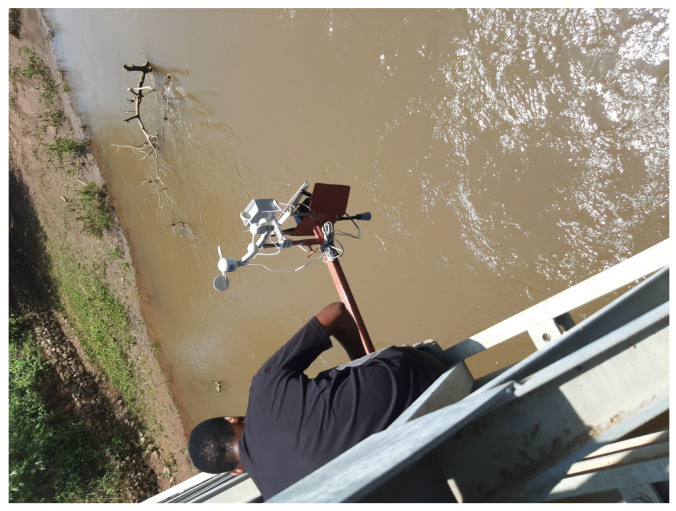
Sensor installation at Kikuletwa bridge.

To identify the optimal locations for installing sensors along the river, a preliminary study was conducted, with a special focus on the riverside villages. The study aimed to gather critical data for the research while also addressing the needs of the Pangani Water Board (PWB), the regulatory authority responsible for overseeing the management of ground and surface water in the region. The study was conducted in collaboration with the PWB, which required a permanent water monitoring system to produce satisfactory water flow and to control its behavior. Based on the feasibility study findings, two locations were identified as optimal for installing sensors: the bridge at Kikavu chini village, and the Kikuletwa bridge (Figure 3) situated at the border of the Kilimanjaro and Manyara regions. These locations are critical for monitoring and recording river levels, and they are essential inputs for the development of an effective flood warning system.

### 3.1. Waspmote Plug&Sense Smart Agriculture PRO

**Figure 4:**
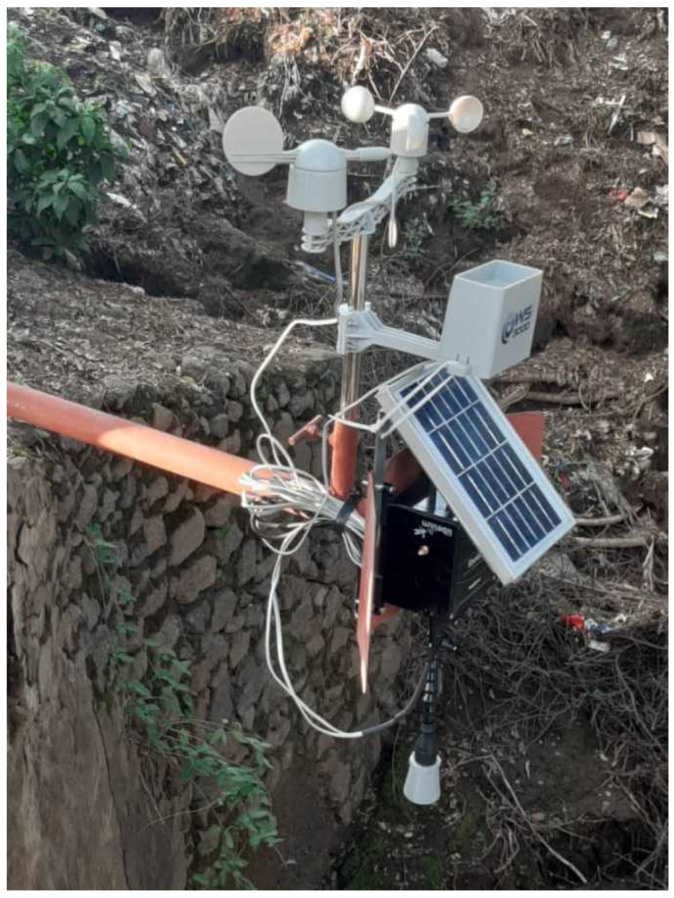
Plug&Sense! Smart Agriculture PRO with all parts connected.

Our study employed the cutting-edge Waspmote Plug&Sense Smart Agriculture PRO, an Agriculture v30 Board, to attach our sensors (as shown in Figure 4). This innovative device enables us to monitor various environmental parameters, including air and soil temperature, humidity, solar radiation, wind speed and direction, rainfall, atmospheric pressure, and more. For our research, we focused on monitoring crucial factors such as river water level, rainfall, and wind speed and direction. The sensor sockets are configured in accordance with the details outlined in Table 1.

Two devices were installed in two different river locations, with each having an ultrasonic sensor for distance measurement, a weather station containing a pluviometer for rainfall measurement, a wind vane for wind direction, and an anemometer for wind speed.

#### 3.1.1. Weather Station

The weather station consists of three different sensors: a wind vane, an anemometer, and a pluviometer. It is connected to Waspmote through six wires that are connected to the terminal block, as can be seen in Figure 4 where the anemometer is connected to the vane through an RJ11 socket.

The specifications for each of the sensors included in the weather station are summarized in Table 2.

#### 3.1.2. Ultrasonic Sensor (MaxSonar^®^ from MaxBotix^TM^)

**Figure 5:**
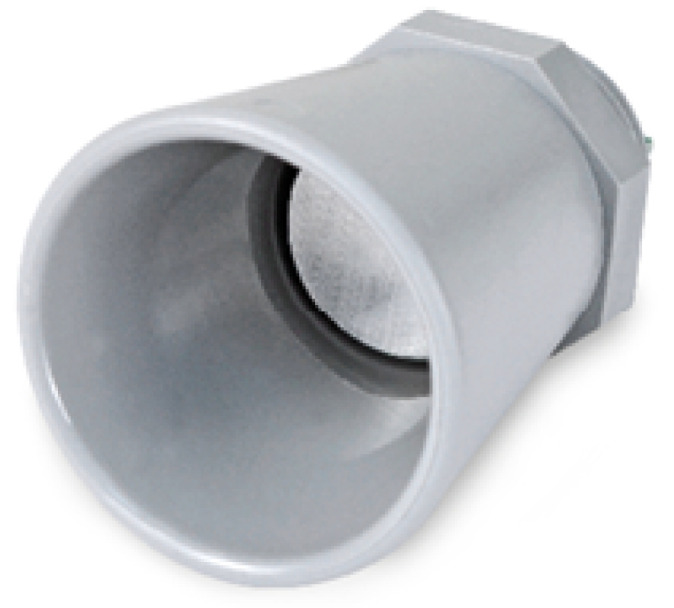
Ultrasonic I2CXL- MaxSonar^®^-MB7040 from Max- Botix^TM^ sensor.

The ultrasonic sensor (Figure 5) measures the distance between itself and the surface of water. It emits ultrasonic waves which rebound in the water, and offers the system with distance between the node and the water. The water level of the river is then calculated. Specifications for the ultrasonic sensor are listed in Table 3.

#### 3.1.3. 4G Module

The Waspmote Plug&Sense is integrated with a 4G radio module for seamless wireless communication, allowing for high-speed connectivity to the LTE, HSPA+, and WCDMA cellular networks. It is specifically designed to work with internet servers, implementing multiple application layer protocols internally to facilitate seamless data transmission to the cloud. Additionally, the device enables HTTP navigation, and the uploading and downloading of diverse contents to web servers, as well as secure connections using SSL certificates, and the setting of TCP/IP private sockets. Moreover, it supports the File Transfer Protocol (FTP), which proves to be beneficial in handling files within an application. By leveraging the power of this advanced technology, our research is able to collect and to transmit accurate data on critical environmental parameters.

#### 3.1.4. Battery and Solar Panel

The devices are equipped with two types of batteries for the OEM line, a 6600 mAh, rechargeable lithium-ion battery (Li-Ion), with 3.7 V nominal voltage and a 52,000 mAh non-rechargeable battery with a 3.4 V nominal voltage. Waspmote is equipped with a control and safety circuit, through which the battery charge current is always made sure to be adequate. On the other hand, in the power sources for the devices, we have a solar panel. Waspmote comes with a rigid solar panel of 7 V, 500 mA which can allow up to 12 V, and the maximum charging current through the solar panel is 300 mA.

### 3.2. River Depth Measurement

**Figure 6:**
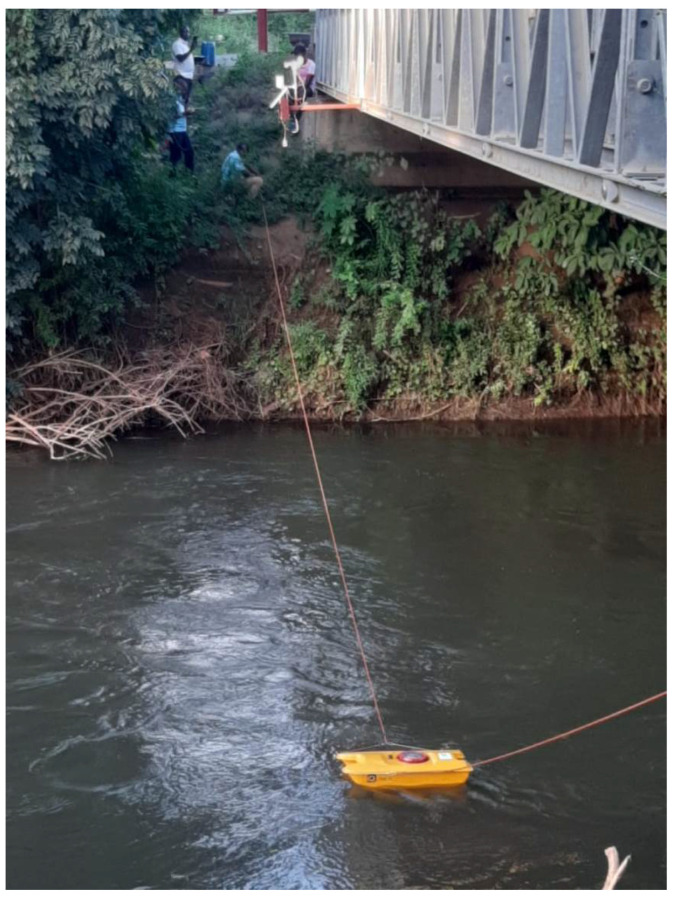
Echo sounding technique employed to determine initial river level.

To establish an initial reference for determining river levels, we conducted measurements of the river depth at both locations. The ultrasonic sensor utilized in our research measures the distance between its location and the water surface. This distance is subsequently transformed into river levels after determining the initial depth using the bathymetry technique. Bathymetry is a widely used technique for measuring water depth in oceans, rivers, or lakes. By applying this technique, we were able to accurately determine the initial depth of the river, which serves as a crucial reference point for subsequent measurements and data analysis (https://education.nationalgeographic.org/resource/bathymetry) (accessed on 12 January 2023). Similar to topographic maps, bathymetric maps use lines to connect points of equal depth of the river. Echo sounding technology was employed (Figure 6). Echo sounding is a bathymetry technique of measuring depth using sonar (https://en.wikipedia.org/wiki/Echo_sounding) (accessed on 12 January 2023).

The sonar (echo sounder) depicted in Figure 6 emits a beam of sound downward to the river floor, commonly referred to as “pings”. The time taken for the sound to travel through the water, reflect off the river floor, and return to the echo sounder provides information about the distance to the river floor. Prior to deploying the sensors, we conducted measurements using the sonar in both locations to determine the river floor depth. By utilizing this advanced technology, we were able to accurately measure the distance between the sonar and the river floor, which proved to be a crucial input in our data collection and analysis.

#### Level Measurement at Both Bridges

The Kikavu bridge is situated at a latitude of −3.44 and a longitude of 37.30, representing the more upstream of the two locations we studied. On the other hand, the Kikuletwa bridge is situated at a latitude of −3.55 and a longitude of 37.31, representing the more downstream of the two locations. The ultrasonic sensor installed at the Kikavu bridge (kkv) was mounted at a height of 765 cm from the surface of water during installation, which coincided with normal flow (the flow during the dry season). In order to establish an initial reference point, we measured the initial river depth at Kikavu, which was found to be 94 cm. By accurately measuring the location and height of the ultrasonic sensor, we were able to collect reliable and precise data on river levels. The total distance from the sensor to the riverbed at Kikavu can now be written as:

(1) $$d_{t}=d_{0}+d_{v}=765+94=859\;\mathrm{cm}$$

**Figure 7:**
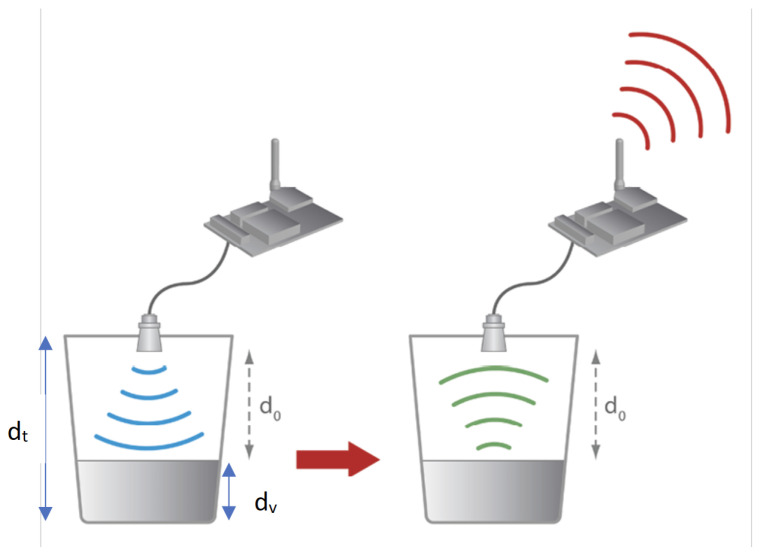
Ultrasound sensor measurement process.

So, to obtain hourly river height from the incoming sensor data, we use the following formula:

(2) $$d_{v}=d_{t}-d_{0}$$

where $d_{v}$, is the river height at Kikavu bridge, $d_{t}$, the distance from the sensor to the riverbed, and $d_{0}$, is the sensor-read distance or the distance from the sensor to the water surface (see Figure 7).

The same procedure was was executed at Kikuletwa bridge with the sensors this time being mounted at a height of 400 cm from the surface of the water. The initial river depth at Kikuletwa was measured at 130.5 cm. The total distance from the sensor to the riverbed at Kikuletwa bridge can now be written as:

(3) $$d_{t}=d_{0}+d_{u}=400+130.5=530.5\;\mathrm{cm}$$

So, to obtain the hourly river height from the incoming sensor data, we use the following formula:

(4) $$d_{u}=d_{t}-d_{0}$$

where $d_{u}$ is the river height at Kikuletwa bridge, $d_{t}$ is the distance from the sensor to the riverbed, and $d_{0}$ is the sensor-read distance or the distance from the sensor to the water surface.

### 3.3. Programming of the Sensor Nodes

**Figure 8:**
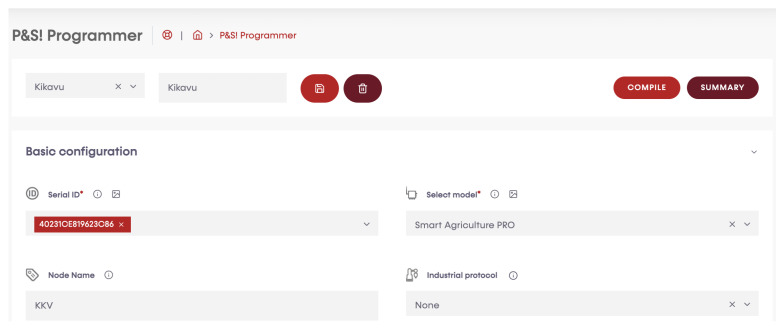
Device selection using serial number.

**Figure 9:**
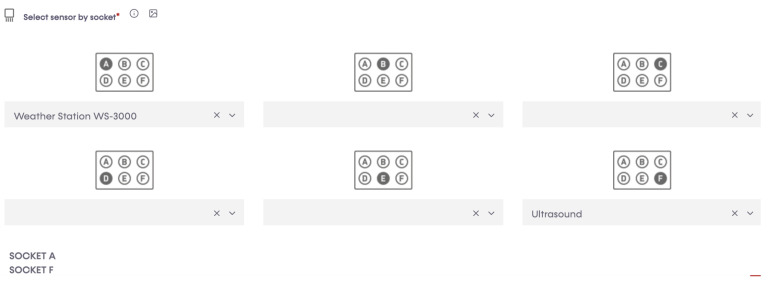
Sensor socket selection.

**Figure 10:**
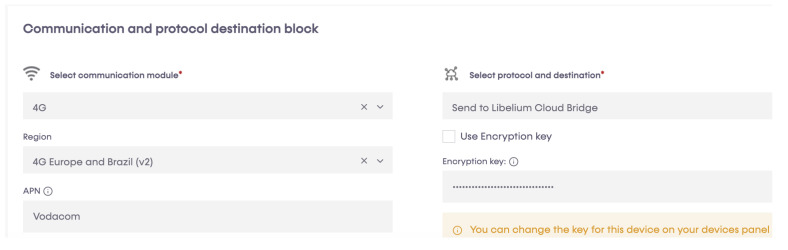
Setting up communication and protocol destination block.

**Figure 11:**
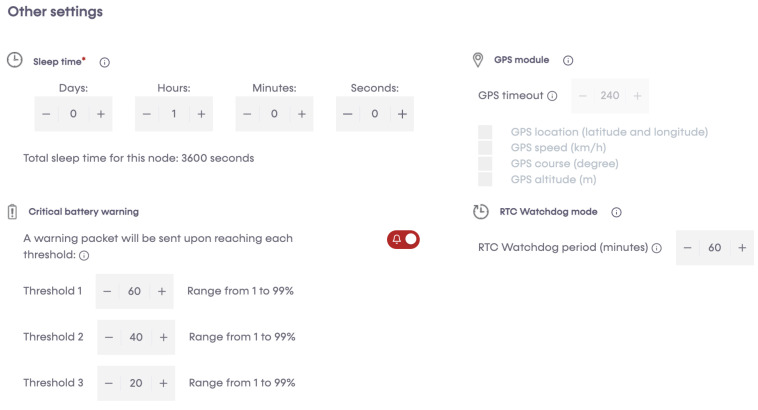
Other settings.

A programming cloud service provided by Libelium has a special application, P&S programmer, where each sensor node has been programmed to send data to Libelium cloud bridge (Figure 8, Figure 9, Figure 10 and Figure 11). Basic configurations for each sensor socket were created through P&S programmer; such configurations include communication and the protocol of the destination block; in this case, 4G was set as a communication module. Other settings include sleep time, set at 3600 s (1 h) for energy efficiency. This is the amount of time the device spends in sleep mode before a new cycle (sensor reading + transmission is performed). Furthermore, critical battery warning setting, where three thresholds (60%, 40%, and 20%) were set. A warning packet is sent upon reaching each threshold. After all the settings, we compiled to create binary files, which are then uploaded to the devices through the Smart Devices Application. Meanwhile, a valid API key and encryption layer functions were transparently provided for each node. A valid API key and encryption functions are a must when programming the sensor nodes using Waspmote IDE. API keys authenticate calls to the Libelium cloud bridge service discussed in Section 3.4.

#### Smart Devices App

The Smart Devices App was one of the most important tools during the process of installing programs to Waspmote Plug & Sense. Binary files generated from the P&S programmer, were uploaded to each of the devices though the Smart Devices App. The latest version of the Java Development Kit (JDK) is a requirement before using the Smart devices App; in our case, during the time of installation, we used Java SE 18. From the Smart Devices App, all the Plug & Sense devices that listed updates to programs were created from there after selecting a firmware that needed to be upgraded.

### 3.4. The Cloud Bridge

The data gathered by the sensor are sent to Libelium cloud bridge where they can be visualized in their raw state, and their status can be checked from time to time. The bridge is a service that allows users to send information from any IoT device to the main worldwide cloud platforms simultaneously, and without having to implement each specific cloud protocol or authentication methodology. In our case, we connected the bridge to Microsoft Azure. The Libelium cloud service has a buffer of limited size that is cleared after that data are sent to the final cloud service. The bridge has three main functionalities:

- Configure cloud connectors,
- Manage devices,
- Configure gateways.

All of these functionalities are oriented to send data from the sensor nodes to the final cloud service.

#### Data Flow

**Figure 12:**
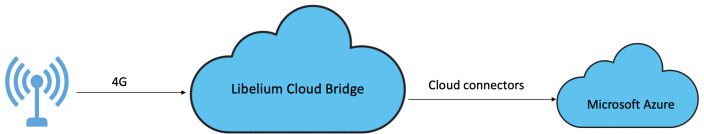
Libelium Cloud Bridge service data flow.

The primary data sources are the sensor nodes, which send data to the cloud service through the Libelium cloud bridge. To facilitate effective data management and analysis, we implemented a comprehensive dashboard within the cloud service, which receives real-time data from the sensor nodes. Our cloud service is powered by Microsoft Azure, a state-of-the-art cloud computing platform, as illustrated in Figure 12. By leveraging the power of cloud-based computing and data storage, we were able to collect and visualize large amounts of data in real-time, providing critical insights into river levels and other environmental parameters.

Sensor nodes, through the 4G communication protocol, send data directly to the Libelium cloud bridge service. The Libelium Cloud bridge service listens to HTTPS requests to receive data from the sensor nodes. Valid requests must comply with the following requirements:

- The sensor node must be registered in the Libelium Cloud (Services Cloud Manager) user account,
- A valid authentication API Key must be associated with the sensor node,
- The integrity of the Libelium Cloud Bridge service encryption layer is respected.

### 3.5. Data Storage and Visualization

**Figure 13:**
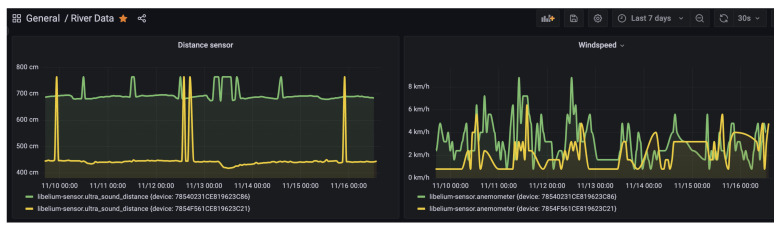
The dashboard on the virtual machine.

At the data receiving end, we deployed a virtual machine with the necessary specifications to handle the high volume of incoming data streams from the sensor nodes. To facilitate efficient and effective data management and analysis, we leveraged the capabilities of InfluxDB, a high-performance time-series database designed for handling large volumes of time-stamped data. Additionally, we utilized Grafana, a powerful open-source visualization platform, to enable real-time data visualization, querying, and analysis (see Figure 13).

The Libelium cloud bridge provided the necessary connectivity between the sensor nodes and the Microsoft Azure cloud connector, a scalable cloud-based service for storing and managing data. We implemented an Azure storage container to securely store the large amounts of unstructured data received from the sensor nodes.

To enable seamless data integration and processing, we developed a custom Python script on the virtual machine that periodically checks for new data files every two hours and inserts them into InfluxDB. This script leverages InfluxDB’s APIs to provide automated and efficient data ingestion, while also enabling us to configure data retention policies to manage our data storage requirements.

## 4. Data Characterization

The data set consists of six variables, measured hourly. The variables are river level in centimeters (cm) measured using an Ultrasound sensor, current hour rainfall (mm), previous hour rainfall (mm/h), and last 24 h rainfall (mm/day), all measured using the pluviometer; wind speed in kilometers per hour (km/h) using the anemometer, and wind direction as the direction by the wind vane. The most important features of these are rainfall and river level, as they can directly state the condition of the river and if there will be any flood risks. Rainfall patterns are the main natural factor affecting water levels, with periods of wet conditions resulting in increasing water levels. On the other hand, wind speed and direction affect rainfall patterns; therefore, in this context, we have water level as a dependent variable, while the rest are independent.

### 4.1. River Levels

**Figure 14:**
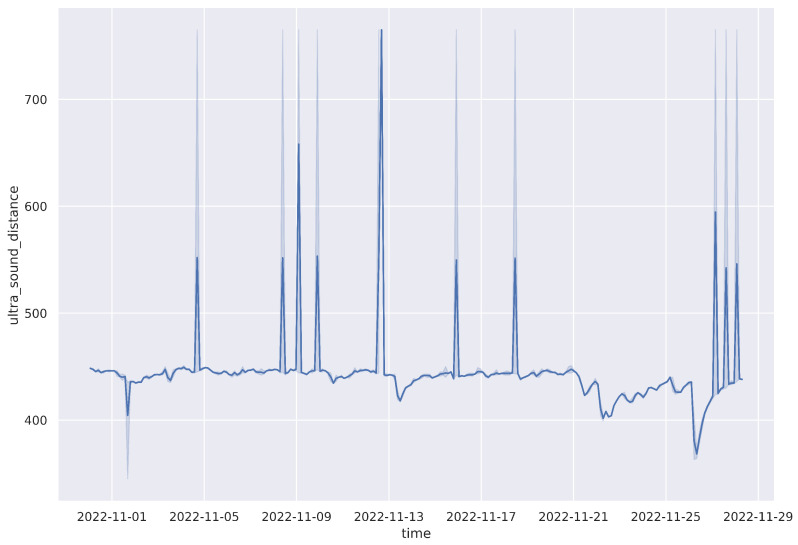
Ultrasound data plot for November 2022 at Kikuletwa bridge.

River water levels are measured hourly in centimeters by the ultrasonic sensor. The river level is then calculated based on the initial water level, recorded at a corresponding point by applying the formulas explained in Section 3.2. Since deployment, there has been an almost constant distance reading, with an average of 444.12 cm (Table 4). The trend for November, 2022 (see Figure 14) is representative of how the situation has been for all other months for that location. This has mainly been due to below-average rainfall in the last two rain seasons all over the country due to a changing climate [23].

### 4.2. Rainfall

**Figure 15:**
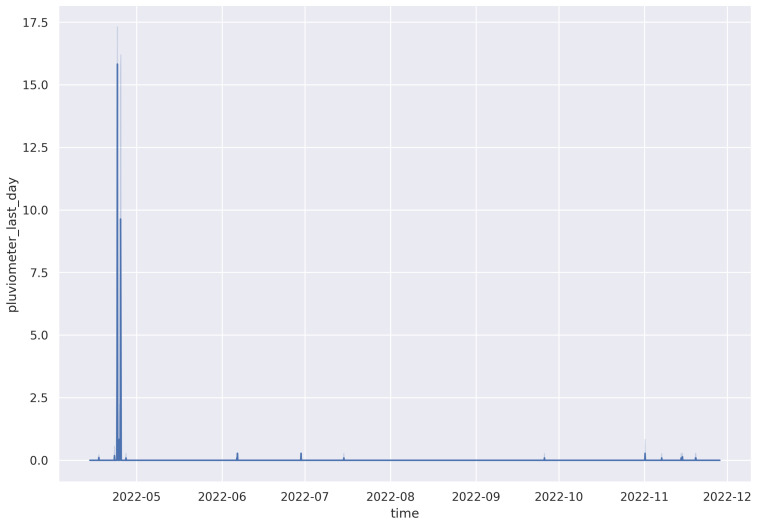
Daily rainfall trend from April to November 2022.

The pluviometer gives three types of readings, rainfall for the current hour in millimeters (mm), previous hour (mm/h), and accumulated rainfall in the last 24 h (mm/day). The pluviometer has a small bucket of approximately 0.28 mm when full. During the measuring process, the switch is closed when the bucket is full, and then it is emptied afterwards. Some characteristics of rainfall data are shown in Table 5, with a corresponding plot (Figure 15) for the whole period since deployment in April 2022.

### 4.3. Wind Speed

**Figure 16:**
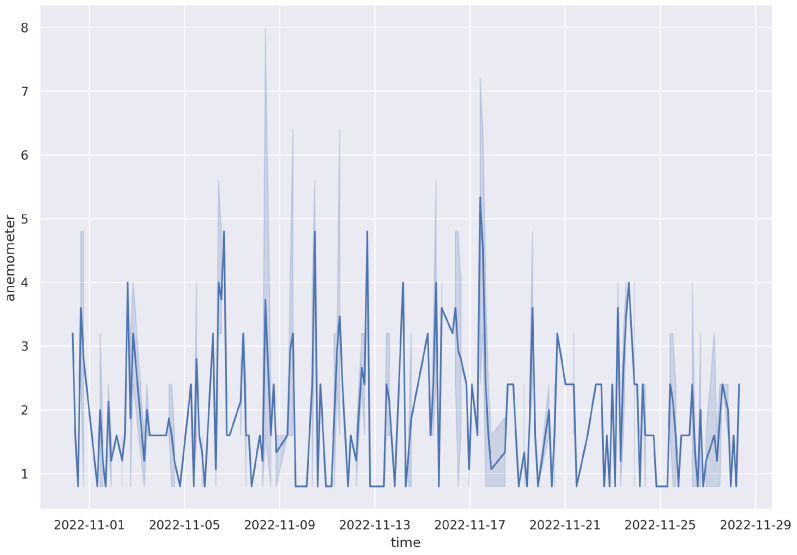
Hourly wind speed data for November 2022.

The anemometer used consists of a read switch that is normally open, and it closes only for a short time when the arms of the anemometer turn to complete a $180^{\circ }$ angle. The reading of the anemometer is a digital signal, whose frequency is proportional to the wind speed in kilometers per hour (km/h). An example of the data from the anemometer for the month of November is shown in Table 6, with a corresponding plot (Figure 16).

### 4.4. Wind Direction

The wind vane measures the direction. Table 7 shows the different values that the equivalent resistance of the wind vane may take, together withe the direction corresponding to each value.

### 4.5. Exploratory Data Analysis

To investigate some relationships among different variables, an exploratory data analysis was performed on a chunk of data collected from April to November 2022. The data came from one of the locations, Kikuletwa bridge. A correlation matrix was one of the tools used to show the correlation coefficients between the variables in the data set. A correlation coefficient ranges from −1 to 1, and indicates the strength and direction of the relationship between the variables.

**Figure 17:**
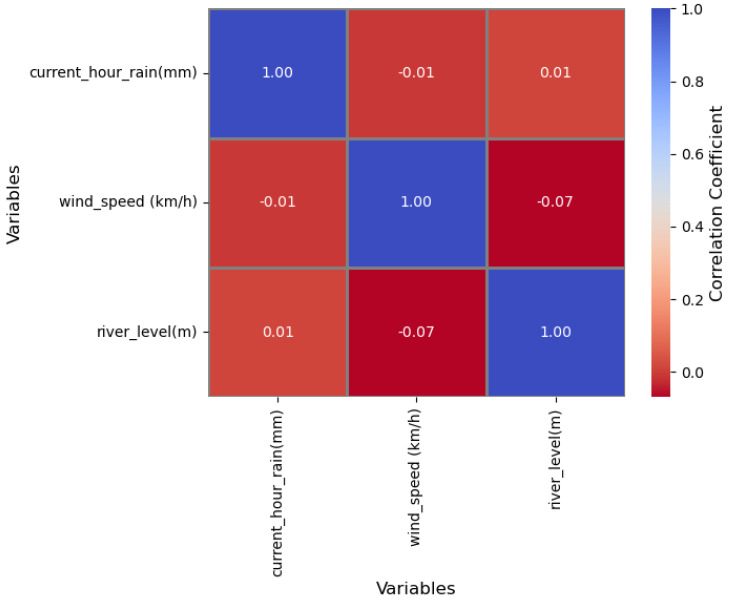
Correlations among rainfall, wind speed, and river levels in one location for the stated period.

Looking at the matrix (Figure 17), we can see that there is a weak positive correlation (0.012) between the current hour rain and the river level. This means that as current hour rain increases, the river level tends to slightly increase as well.

There is also a weak negative correlation (−0.007) between the current hour rain and wind speed, meaning that as the current hour rain increases, the wind speed tends to slightly decrease. Similarly, there is a weak negative correlation (−0.069) between the wind speed and the river level, indicating that as the wind speed increases, the river level tend to slightly decrease.

Overall, the correlations between these three variables are weak and suggest that there is no strong linear relationship between them. However, for the chunk of data selected, it is important to note that correlation does not necessarily imply causation, and further analysis would be needed to understand the underlying relationship between these variables. Histograms for the selected continues variable were also plotted to show the distribution of main variables.

**Figure 18:**
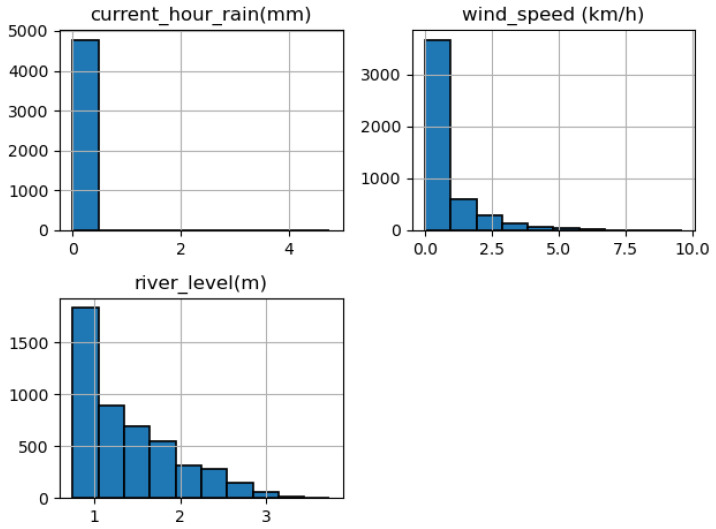
Histograms of the selected continues variables for the time given.

**Figure 19:**
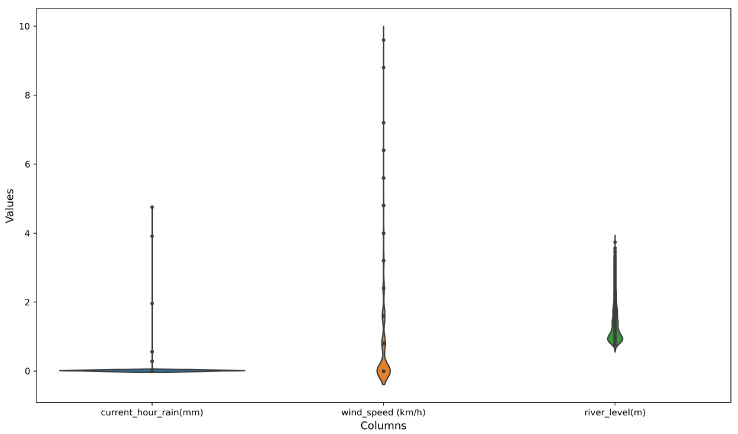
Distribution of some selected continues variables.

In the case of current rain, the histogram being concentrated at 0 and just above suggests that most of the time there is no rain, or very little rain. This is supported by the mean value being very close to 0, and the minimum value being 0, which of course, is the reality for Tanzania in 2022, with severely below-average rainfall [23]. That was subsequently observed through relatively low river flows, as suggested by the river level flowing just above the zero mark most of the time (Figure 18). This was also in line with a violin plot (Figure 19) with a concentration of values at 0 and just above, while there were narrower and taller shapes on the right representing a smaller number of non-zero values. The thickness of the violin plot at any given point represents the density of the data points at that point, so the violin plot was the thickest at around 0 and just above.

## 5. Discussion

The design and implementation of sensor-based river monitoring systems has gained considerable attention in recent years due to their potential to provide accurate and timely flood early warnings [24]. This study presented a solution that aims to improve the quality of the data used for flood early warning systems, which is crucial for the success of such systems.

As emphasized in the paper, the accuracy and completeness of the data is paramount in determining the effectiveness of the warnings [25]. Hence, the solution proposed focuses on gathering data that are of high precision, accuracy, and accessibility to all stakeholders [26]. The authors highlight the importance of river basins as essential resources that support human and animal life [27], and emphasize the potential of the monitoring system to provide critical information for water management processes such as floodwater utilization for irrigation and other economic activities [28].

Recent studies have shown that the integration of remote sensing data and machine learning algorithms can significantly enhance the accuracy and reliability of flood early warning systems [29,30]. In this regard, the authors suggest that the proposed monitoring system can be improved by incorporating these approaches in future research. Additionally, the use of real-time monitoring systems and mobile technologies can improve the efficiency and speed of the disaster response, as discussed in previous research [31].

However, onsite setup of the monitoring devices presents challenges in terms of the quality of data collected. Several sources of noise were identified that must be put into consideration during further analysis of the data set. For instance, the untimely recharging of the internet data can lead to gaps in the data, which can be easily identifiable in the data set since it mostly occurs in the last or first days of the month. In addition, the malfunctioning of the devices can result in out-of-range readings, which can be seen from ultrasonic distances that are beyond the possible distance that can be recorded.

The solution presented in the paper aligns with the United Nations Sustainable Development Goal 6, which aims to ensure the availability and sustainable management of water and sanitation for all [32]. The authors suggest that the proposed monitoring system can contribute to achieving this goal by providing critical information that can support better decision-making in water resource management and allocation.

Furthermore, the solution addresses the challenges of monitoring river floods in the Kikuletwa River. The proposed monitoring system has the potential to improve the quality of data collected and provide critical information for water management processes, while aligning with the United Nations Sustainable Development Goal 6. Future research can build upon the proposed solution by incorporating remote sensing data and machine learning algorithms to enhance the accuracy and reliability of flood early warning systems.

### 5.1. Flood Prediction Context

The data generated from this experiment are hourly multivariate time series data, which contain multiple variables observed over a period of time. Machine learning algorithms such as AutoRegressive (AR), Autoregressive Integrated Moving Average (ARIMA), and deep learning algorithms such as Recurrent Neural Networks (RNN) in particular, are better suited for time-series data, such as river water levels and flow rates [33]. A key attribute of recurrent neural networks is their ability to persist information, or cell state, for use later in the network [34]. This makes them particularly well suited for the analysis of temporal data that evolve over time. By analyzing the data set, we can identify patterns and trends that can be used to develop accurate and reliable flood forecasting models. The use of AI/ML-based forecasting methods can further enhance the accuracy of these models by enabling them to learn from historical data and to adapt to changing conditions in real-time.

Another approach is to use ensemble-based methods, such as random forests and gradient boosting, which combine the predictions of multiple models to produce a more accurate forecast. These methods are particularly useful when dealing with complex and nonlinear relationships between the input variables and the target variable [35,36].

Looking again at the simple exploratory data analysis explained in Section 4.5, which of course is just one aspect of he analysis that needs to be considered when building a flood prediction model, we obtain some useful insights. On one hand, these insights suggest that the current hour rain and wind speed may not be strong predictors of river level, and they may not be very useful for flood prediction. However, the river level appears to be relatively more spread out, suggesting that it may be a more useful variable for modeling flood events. On the other hand, any conclusion drawn from these plots should be considered within the context of the specific data and time period analyzed. It would be important to analyze data over a longer period of time to draw more robust conclusions about flood prediction. Additionally, incorporating other variables such as precipitation patterns, land use, and hydrological characteristics of the river would also be important for developing an accurate flood prediction model [37].

In this view, combining the power of AI/ML-based forecasting methods with the rich and diverse data set generated by the sensor-based river monitoring system, effective flood prediction models can be developed to help prevent or minimize the devastating effects of flooding.

### 5.2. Beyond Flood Prediction

Firstly, the data set generated from the sensor-based river monitoring system for the Kikuletwa river floods can be used to improve water management and irrigation practices. By analyzing the data on water levels and flow rates, water managers can make informed decisions on when and how much water to release from reservoirs or dams for irrigation, and how to allocate water resources among different users [38]. This can help ensure that water resources are used efficiently and sustainably, and it can help to prevent conflicts over water usage.

Secondly, the data set can be used to monitor the health and biodiversity of the river ecosystem. By analyzing the data on water quality, temperature, and other environmental parameters, scientists and conservationists can assess the impact of human activities on the ecosystem, identify areas of concern, and develop conservation strategies to protect and to restore the ecosystem. This can help to ensure the long-term sustainability of the river ecosystem, and it can help to promote environmental stewardship and awareness.

Furthermore, the data set generated from the sensor-based river monitoring system can be used for research and educational purposes. Researchers can use the data to develop models and simulations of river systems, test hypotheses, and discover new insights into the workings of rivers. Educators can use the data to teach students about the importance of rivers and their role in the environment, and to promote environmental awareness and stewardship. This can help advance our understanding of river ecosystems and their importance, and can help educate future generations of environmental leaders and stewards.

Moreover, the data set generated from the sensor-based river monitoring system can be used for early drought detection by continuously monitoring the water levels and flow rates in the river [39]. When water levels remain low for an extended period of time, it can be an indication of a drought, even before the effects of the drought are visible on the surface. By detecting these early warning signs of drought, officials can take proactive measures to mitigate the effects of the drought and reduce the impact on communities and the environment. This can include implementing water conservation measures, such as restrictions on water usage, encouraging the use of drought-resistant crops, and implementing water-efficient irrigation techniques. Early drought detection can also help water managers to better allocate water resources among different users, such as farmers, industries, and households. Overall, the use of the data set generated from the sensor-based river monitoring system for early drought detection can help prevent conflicts over water usage, ensure fair and equitable water resource management, and promote sustainable water management practices.

Finally, the data set can be used to aid disaster response and recovery efforts in the event of a flood or other natural disaster. By providing real-time data on river conditions, the system can help emergency responders to identify areas of concern, allocate resources, and make informed decisions on how to respond to the disaster. After the disaster, the data set can be used to assess the extent of the damage, identify areas that need to be prioritized for recovery, and track the progress of recovery efforts over time. This can help to ensure that the disaster response and recovery efforts are efficient and effective, and it can help communities to recover more quickly from natural disasters.

Overall, by leveraging the data set generated from the sensor-based river monitoring system for a range of purposes beyond flood warning systems, we can better understand, manage, and protect the complex systems that make up our natural environment.

### 5.3. Potential Limitations of the Approach

One potential limitation of the proposed solution of using wireless sensor networks for river level monitoring is that it relies on the availability of reliable and stable wireless connectivity. If there are disruptions in the wireless network, or if the sensors are located in areas with poor network coverage, data transmission and collection may be compromised.

Another limitation is that the accuracy of the data collected by the sensors can be affected by various environmental factors, such as interference from other electronic devices, changes in water flow patterns, and changes in water temperature. Therefore, it is important to regularly calibrate and maintain the sensors to ensure that they are providing accurate and reliable data.

Furthermore, the cost of implementing and maintaining a wireless sensor network can be relatively high, which may limit its scalability and adoption, particularly in under-resourced areas with limited funding and human capacity. Additionally, there may be challenges in terms of ensuring the security and privacy of the collected data, as well as the reliability of the sensor network over time.

Overall, while wireless sensor networks hold great potential for improving river level monitoring and flood prediction, there are still some limitations and challenges that need to be addressed in order to fully realize their benefits.

## 6. Conclusions

In conclusion, the implementation of a multi-model, sensor-based river monitoring system for the Kikuletwa river floods has demonstrated significant potential for providing accurate and timely data on river conditions, which is essential for disaster prevention and mitigation. The system combines sensors and traditional methods to capture real-time data on water levels, flow rates, and other key parameters, laying the groundwork for developing machine learning models for flood prediction in the future. The main benefits of this sensor-based system include its ability to provide continuous and reliable data on river conditions during extreme weather events, its scalability and flexibility for easy deployment and customization, and its potential for enhancing machine learning-based flood prediction accuracy. This study makes several important contributions to the field:

- A multi-model implementation that supports the collection and aggregation of six weather parameters, complementing existing local weather stations and enhancing the region’s resilience to extreme weather event prediction.
- The development of a continuous data set on river characteristics, which can be used to improve flood resilience and water management strategies in the context of changing climate patterns.
- The introduction of a mechanism to define river thresholds, which are a key input in anomaly detection machine learning models for flood prediction and management.

By using advanced technologies such as sensors, cloud-based data storage and analysis, and machine learning algorithms, this study lays the foundation for future research and development in the field of machine learning for flood prediction. The successful implementation of the sensor-based river monitoring system for the Kikuletwa river floods, along with the significant contributions made in this study, can help water management officials to develop more effective and sustainable strategies for managing rivers and preventing floods, ultimately contributing to the broader goal of achieving sustainable development and resilience to climate change, as outlined in the United Nations’ Sustainable Development Goals (SDGs).

Building upon the achievements of this study, future research should focus on the integration and optimization of machine learning algorithms to further improve flood prediction and management. Collaboration between researchers, local communities, and water management authorities will be critical to ensure that these advanced technologies are effectively applied and adapted to the specific needs and contexts of diverse regions. In addition, attention should be given to addressing potential challenges in scaling and customizing the system, such as limited resources, connectivity issues, and the need for localized adaptations, to ensure the sustainability and long-term successes of these efforts.

Moreover, the successful implementation of this sensor-based river monitoring system can serve as a model for other regions facing similar challenges related to flood management and environmental monitoring. By sharing knowledge, experiences, and best practices gained from the Kikuletwa river case study, stakeholders can collaborate to develop and deploy contextually appropriate solutions in other vulnerable areas. This collaborative approach will not only help in addressing immediate flood risks, but it also contributes to the global effort of building resilience to climate change and fostering sustainable development.

## Figures and Tables

**Table 1:** Sensor sockets configuration.

Sensor Socket	Parameter	Reference
A	Weather station WS-3000 (anemometer + wind vane + pluviometer)	9256-P
F	Ultrasound (distance measurement)	9246-P

**Table 2:** Weather station sensor specifications.

Sensor	Specifications
Anemometer	Sensitivity: 2.4 km/h/turn, Wind Speed Range: 0∼240 km/h, Height: 7.1 cm, Arm length: 8.9 cm, Connector: RJ11
Wind vane	Height: 8.9 cm, Length: 17.8 cm, Maximum accuracy: 22.5${}^{\circ }$, Resistance range: 688 Ω∼120 kΩ
Pluviometer	Height: 9.05 cm, Length: 23 cm, Bucket capacity: 0.28 mm of rain

**Table 3:** Weather station sensors specifications.

Sensor	Specifications
Ultrasonic	Operation frequency: 42 kHz, Maximum detection distance: 765 cm, Interface: Digital Bus, Power supply: 3.3∼5 V, Consumption: 2.1 mA (powered at 3.3 V) to 3.2 mA (powered at 5 V), Consumption (peak): 50 mA (powered at 3.3 V) to 100 mA (powered at 5 V), Usage: Indoors and outdoors (IP67)

**Table 4:** Ultrasound distance characteristics for November 2022 at Kikuletwa bridge.

Characteristic	Ultrasound Distance
Count	654
Mean	444.12
Standard Deviation	45.62
Minimum	345
25%	435.25
50%	442
75%	445
Max	765

**Table 5:** Characteristics of rainfall data from one location.

Characteristic	Daily Rainfall
Count	4871
Mean	0.022
Standard Deviation	0.537
Minimum	0.00
25%	0.00
50%	0.00
75%	0.00
Max	17.323

**Table 6:** Characteristics of wind-speed data from one location.

Characteristic	Hourly Wind Speed (km/h) for November 2022
Count	304
Mean	2.06
Standard Deviation	1.32
Minimum	0.80
25%	0.80
50%	1.60
75%	2.40
Max	8.00

**Table 7:** Wind direction value identification.

Direction (Degrees)	Resistance (k)	Voltage (V)	Identifier
0	33	2.53	SENS_AGR_VANE_N
22.5	6.57	1.31	SENS_AGR_VANE_NNE
45	8.2	1.49	SENS_AGR_VANE_NE
67.5	0.891	0.27	SENS_AGR_VANE_ENE
90	1	0.3	SENS_AGR_VANE_E
112.5	0.688	0.21	SENS_AGR_VANE_ESE
135	2.2	0.59	SENS_AGR_VANE_SE
157.5	1.41	0.41	SENS_AGR_VANE_SSE
180	3.9	0.92	SENS_AGR_VANE_S
202.5	3.14	0.79	SENS_AGR_VANE_SSW
225	16	2.03	SENS_AGR_VANE_SW
247.5	14.12	1.93	SENS_AGR_VANE_WSW
270	120	3.05	SENS_AGR_VANE_W
292.5	42.12	2.67	SENS_AGR_VANE_WNW
315	64.9	2.86	SENS_AGR_VANE_NW
337.5	21.88	2.26	SENS_AGR_VANE_NNW

## Data Availability

The data presented in this study are available on request from the corresponding author. The data are not publicly available due to active research going on, and still need to be kept private for the moment. It is, however, expected that the data generated from this study will be made publicly available at the end of the main research period.

## Abbreviations

The following abbreviations are used in this manuscript:

JDK	Java Development Kit
USB	Universal Serial Bus
CSV	Comma Separated Values
OEM	Original Equipment Manufacturer
AI	Artificial Intelligence
ML	Machine Learning
PWB	Pangani Water Board
HTTP	Hyper Text Transfer Protocol
TCP/IP	Transfer Control Protocol/Internet Protocol
FTP	File Transfer Protocol
API	Application Programming Interface
IDE	Integrated Development Environment
